# Hiding in plain sight—*Euplokamis dunlapae* (Ctenophora) in Norwegian waters

**DOI:** 10.1093/plankt/fbab012

**Published:** 2021-03-17

**Authors:** Sanna Majaneva, Halldis Ringvold, Ellie Johansen, Mari-Ann Østensen, Aino Hosia

**Affiliations:** Department for Arctic and Marine Biology, UiT The Arctic University of Norway, Tromsø NO-9037, Norway; Trondhjem Biological Station, Department of Biology, Norwegian University of Science and Technology, Trondheim NO-7491, Norway; Sea Snack Norway, Bergen NO-5841, Norway; Trondhjem Biological Station, Department of Biology, Norwegian University of Science and Technology, Trondheim NO-7491, Norway; Trondhjem Biological Station, Department of Biology, Norwegian University of Science and Technology, Trondheim NO-7491, Norway; Department of Natural History, University Museum of Bergen, University of Bergen, Bergen NO-5020, Norway

**Keywords:** *Euplokamis*, Ctenophora, Cydippida, coastal waters, North Atlantic, Arctic

## Abstract

Cydippid ctenophores of genus *Euplokamis* have been rarely reported from the north-east Atlantic in the scientific literature. The conspicuous lack of previous records is likely attributable to methodological constraints detrimental to sampling ctenophores, including the use of plankton nets and preservation of samples as well as poor identification literature and a lack of taxonomic expertise on gelatinous zooplankton. Here, we have compiled published and novel records as well as documented diver observations, of *Euplokamis* spp. in Norwegian waters. Despite scant earlier reports, our data suggest that the genus *Euplokamis* is widely distributed and relatively common along the entire Norwegian coast, including Svalbard. *Euplokamis* was recorded from samples taken from several hundred meters depth to surface, from fjords as well as offshore. Most of the observations reported in this study are from the period between April and July, whereas specimens have been found nearly throughout the year. Specimens from Norwegian waters were morphologically most similar to *Euplokamis dunlapae*, and conservative 18S rDNA sequences of some specimens had a 100% match with an *E. dunlapae* specimen from Friday Harbor, USA, the type locality for the species. However, the morphological and molecular variation of *Euplokamis* demonstrates the need for systematic global sampling of multiple individuals of many ctenophore species.

## INTRODUCTION

**Fig. 1:**
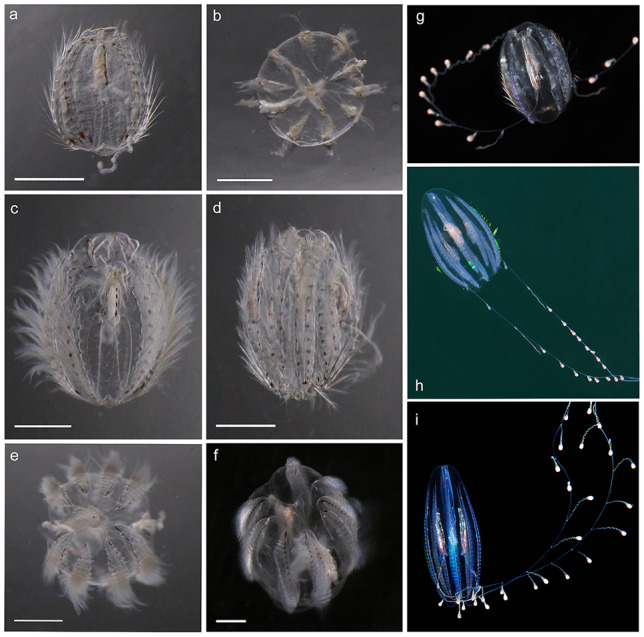
*Euplokamis* cf. *dunlapae* of various sizes from Norwegian waters. Scale bars 1 mm. Net caught specimens photographed live with a stereomicroscope (**a**–**f**): (a) tentacular plane (MT614573), (b) oral view (Fanafjorden 03.03.2016), (c) tentacular plane (MT614566), (d) stomodaeal plane (MT614589), (e) aboral view (MT614566) and (f) aboral view (MT614577). Undamaged specimens show the change in general body shape with increasing size (**g**–**i**): (g) small surface caught specimen, Espegrend 21.4.2015, photo by Fredrik Pleijel, (h) mid-sized specimen, photographed *in situ* by Nils Aukan and (i) large specimen photographed *in situ* by Erling Svensen. Refer to Table I for observation details.

The monotypic ctenophore family Euplokamididae Mills, 1987 (previously Euplokamidae) is characterized by tentacle side branches containing striated muscle, a unique feature within the phylum Ctenophora (Mills, 1987; Mackie *et al.*, 1988). The widely spaced coiled tentilla, rapidly discharged upon contact with prey (Mackie *et al.*, 1988), have a characteristic droplet-like appearance that allows easy identification of live specimens to genus level (Fig. 1).

The World Register of Marine Species (WoRMS, accessed 24 January 2020) lists six species of *Euplokamis* as valid: *Euplokamis crinita* (Moser, 1909), *Euplokamis dunlapae*  Mills, 1987, *Euplokamis evansae*  Gershwin *et al.*, 2010, *Euplokamis helicoides* (Ralph and Kaberry, 1950), *Euplokamis octoptera* (Mertens, 1833) and *Euplokamis stationis* (Chun, 1879). The genus is poorly represented in modern identification literature: The only existing key to species (excluding *E. evansae*) is by Mills (1987), while Gershwin *et al.* (2010) present a table comparing diagnostic characters. It is worth noting that the validity of several of the *Euplokamis* species mentioned in these sources has been questioned. *Euplokamis brunnea*, included in the key by Mills (1987), has been found to lack the striated muscle characteristic of the genus and has thus been moved to the genus *Pleurobrachia* (Mills, 1987). The *E. crinita* specimens described by Moser (1909) were all small (<4 mm) and exhibited characters that suggest they may have been juveniles of one of the other species (Mills, 1987). Mills (https://faculty.washington.edu/cemills/ActaErrata.html, accessed 13 February 2020) also suspects that *E. octoptera* may in fact be a synonym for *Mertensia ovum* (Fabricius, 1780) and comments that the tentacles of *E. evansae* do not seem to justify its inclusion in the genus *Euplokamis* (Mills, 1998-present), where it was provisionally placed by Gershwin *et al.* (2010).

In addition to the doubts regarding the validity of several *Euplokamis* species and meager identification literature, molecular identification of *Euplokamis* is currently of limited value: of the gene regions commonly used for species identification, only 18S and ITS1 sequences from five specimens are available in public repositories (GenBank, BOLD, SILVA; accessed 17 January 2020). Only one of these records is identified to the species level as *E. dunlapae* (MF599307 for 18S) from the north-east Pacific, while the remaining four are listed as *Euplokamis* sp. (HE805698; HE647719; HE805699; HF912430—containing complete or partial 18S and ITS1).

Of all the *Euplokamis* species, only *E. dunlapae* and *E. stationis* are reported in scientific literature with any frequency. The species with the most mentions in the literature is *E. dunlapae*, which has its type locality in Friday Harbor, Washington, and is frequently observed in the east Pacific (Mills, 1987; Mackie *et al.*, 1988). *Euplokamis dunlapae* has also been recorded in the north-west Atlantic in the 1990’s (Mills, 1995). *Euplokamis stationis* was originally described from the Bay of Naples and has since also been observed in the Alboran Sea in April 1991 (Mills, 1996; Haddock and Case, 1999). Outside the Mediterranean, JAMSTEC reports *E. stationis* from Sagami Bay, Japan (*E. stationis*, in GBIF Secretariat, 2019). Of the remaining, less frequently reported *Euplokamis* species, *E. crinita* (previously described as *Pleurobrachia crinita*) was described based on several specimens collected near Greenland (Mortensen, 1912), while *E. octoptera* was described from Pacific material from the southern coast of Chile and the Bering Strait region. *Euplokamis evansae* is currently assumed to be endemic to Tasmanian waters (Gershwin *et al*., 2010) and *E. helicoides* to New Zealand (Mianzan *et al.*, 2009).

The two most commonly observed species are also the largest in the genus *Euplokamis*. Both are elongate in form: *E. dunlapae* grows up to ca. 20 mm, has an ovate shape and is slightly flattened in the stomodaeal plane, while *E. stationis* has a reported maximum size of ca. 25 mm and is cylindrical in shape (Mills, 1987, 2020; Mills and Haddock, 2007). Comb rows of *E. dunlapae* extend two-third to three-fourth of the body length, while the comb rows of *E. stationis* extend nearly from pole to pole. The orientation of the tentacle sheaths, found midway between the stomodeum and the outer body surface, also differs in the two species, with *E. stationis*’s tentacle sheaths oriented obliquely and *E. dunlapae*’s parallel to the stomodeum.

The scientific literature contains only a few, relatively recent mentions of *Euplokamis* sp. from Norway or the north-east Atlantic. The only report down to species level, as *E. dunlapae*, stems from the Remotely operated underwater vehicle (ROV) images from the Oceana North Sea research expedition in 2016 and 2017 (Álvarez *et al.*, 2019). Generally, specimens are only identified to the genus level. Granhag *et al.* (2012) provided the first observations of the genus in Swedish waters, and also included a personal communication from P. R. Flood and U. Båmstedt, stating that *Euplokamis* sp. has previously been caught by net and observed with submersibles along the west coast of Norway. Majaneva and Majaneva (2013) reported that net caught *Euplokamis* sp. from the Svalbard waters, while Licandro *et al.* (2015), P. Licandro and A. Hosia, personal communication, reported catching *Euplokamis* sp. in the Norwegian Sea. Relying on these published observations alone would seem to imply that the genus is rather scarce, at least in the north-east Atlantic waters. However, a quick search online reveals a number of underwater images identifiable as *Euplokamis* spp., taken by the divers in Norwegian waters, and we also frequently encounter the genus in our net samples taken along and off the Norwegian coast. Video-transects filmed during a 2018 cruise to the southern Norwegian Sea also showed *Euplokamis* spp. to be a common midwater gelatinous predator in the study area (Neitzel *et al*., personal communication).

The aim of the current paper is to document and provide the first comprehensive overview of the occurrence of the genus *Euplokamis* in Norwegian waters. To do this, we have compiled data from all available sources, including our own hitherto unpublished observations, more detailed information on the previously recorded observations by P. R. Flood and U. Båmstedt (Granhag *et al.*, 2012) as well as Licandro *et al.* (2015) and photographs from diver observations. We also provide18S rDNA sequences for several *Euplokamis* specimens from Norwegian waters as well as 18S rDNA intra- and intertaxon divergences [Kimura-2-parameter (K2P) and p-distances] of cydippid ctenophores common in the study area.

## MATERIAL AND METHODS

### Sampling

Ctenophores were collected during several research cruises to various locations along the Norwegian coast, from North Sea to the north of Svalbard, between 2009 and 2018. Sampling was conducted using various nets, including MultiNet (Hydrobios, Kiel, equipped with five closing nets, mesh size 180 μm, opening 0.25 m^2^), WP2 nets (UNESCO, 1968; mesh size 180 μm, opening 0.25 m^2^), modified WP3 nets (non-filtering cod-end, mesh size 780 or 1000 μm, opening 1 m^2^) and a MIK net (mesh size 1.5 mm, filtering cod end, opening 3.15 m^2^), either as a part of regular zooplankton sampling or sampling specifically targeting gelatinous zooplankton. Additional specimens were collected with beakers and dip nets from the surface. As ctenophore sampling during this 10-year period contained dozens of net samples from multiple locations, and only samples containing specimens morphologically identified as genus *Euplokamis* were included into this study. Detailed information on the gear used, location and sampling date is provided in Table I.

**Table I:** Observations of *Euplokamis* spp. from Norwegian and adjacent waters. Observations with specimens sequenced for this study in bold. *Mertensia ovum* specimens’ sequences for this study also listed

Collection date	Locality	Latitude	Longitude	Sampling gear	Sample depth (m)	Sequence ID	Fig. 3 ID	Reference
05 July 1999	Sognefjorden	61.4588	7.5407	WP2	30–400			Flood and Båmstedt, personal communication
22 May 2003	Herdlefjorden	60.5049	5.1883	WP2	0–50			Flood and Båmstedt, personal communication
11 July 2004	Herdlefjorden	60.5184	5.1430	WP2				Flood and Båmstedt, personal communication
26 October 2004	Sognefjorden	61.1031	5.1958	WP3	0–640			Flood and Båmstedt, personal communication
29 October 2004	Osterfjorden	60.5556	5.3668	ROV video				Flood and Båmstedt, personal communication
30 April 2007	Ålesund	62.4559	6.0562	Diver observation				Kåre Telnes
01-01-2010	Kongsfjorden, Svalbard	78, 9 322	11.9057	Diver observation	surface			Geir Johnsen
**2009–2011**	**Kongsfjord, Svalbard**	**78.9861**	**11.1621**	**Multinet, MIK- net**		**HF912430, MT614564**	**A**	Majaneva and Majaneva (2013) **, this study**
**29 October 2010**	**Ytre Skorpo**	**59.9300**	**5.7700**	**Juday 90 μm**	**0–60**	**MT614565**	**B**	**Tone Falkenhaug/IMR**
2011	Gullmarsfjorden, Släggö, Alsbäck and Kristineberg, Sweden	*	*	WP3 & beakers	Surface, 100–110	HE647719, HE805699 HE805698, **MT614574, MT614583, MT614575, MT614576**	C—F	Granhag *et al.*, 2012
01 May 2011	Hottane, Averøy	63.0438	7.3808	Diver observation				Nils Aukan
10 May 2012	Nordsjø	59.2832	4.6685	WP2				This study
11 September 2012	Korsfjorden	60.1846	5.1960	WP3 750 μm				This study
27 April 2013	Klubba, Kristiansund	63.1116	7.7375	Diver observation				Nils Aukan
02 May 2013	Rongesundet, Øygarden	60.4988	4.9332	Diver observation				Anders Schouw
03–12 May 2013	North-west of Norwegian coast	62.4167	5.0731	Mocness	0–25, 25–50, 50–100			Licandro *et al.*, 2015, P. Licandro and A. Hosia, personal communication
21 April 2015	Raunefjorden	60.2697	5.2291	Dip net	surface			This study
24 August 2015	Nordaustlandet, Svalbard	81.9322	15.6797	Multinet	500–1 000			This study
03 March 2016	Fanafjorden	60.2473	5.2869	WP3 750 μm	0–126			This study
12 April 2016	Utsira	59.2833	4.9312	WP3 1 000 μm	0–100			This study
**28 April 2016**	**Fanafjorden**	**60.2473**	**5.2869**	**WP3 750 μm**	**0–130**	**MT614577**	**G**	**This study**
14–15 May 2016	Arboretet, Bergen	60.2557	5.2804	Dip net	Surface			This study
09 July 2016	Isfjord, Svalbard	78.2267	14.1147	WP3 1 000 μm	0–225			This study
**07 September 2016**	**Svalbard**	**80.71683**	**15.552167**	**WP2**	**0–960**	**MT614590**	**H**	**This study**
22 March 2017	Egersund	58.8983	5.5508	Diver observation				Erling Svensen
**06 April 2017**	**Fanafjorden**	**60.2473**	**5.2869**	**WP3 750 μ**	**0–130**	**MT614579, MT614589, MT614582**	**I—K**	**This study**
**06 April 2017**	**Raunefjorden**	**60.2573**	**5.1393**	**WP3 750 μ**	**0–240**	**MT614566, MT614573**	**L—M**	**This study**
06 April 2017	Korsfjorden	60.1846	5.1960	WP3 750 μm	0–665			This study
09 April 2017	Kvalvik fort, Frei	63.1015	7.9005	Diver observation				Nils Aukan
16 April 2017	Gjeslingan, Smøla	63.2290	7.7893	Diver observation				Nils Aukan
23 April 2017	Seivika, Kristiansund	63.1107	7.8731	Diver observation				Nils Aukan
03 May 2017	Egersund	58.8983	5.5508	Diver observation				Erling Svensen
04 July 2017	Egersund	58.8983	5.5508	Diver observation				Erling Svensen
04 July 2018	Ytterøya, Trondheimsfjord	63.7605	11.1125	WP3 1 000 μm	0–100			This study
29 August 2018	Stjørnfjorden, Trondheimsjord	63.7803	9.9684	WP3 1 000 μm	0–100			This study
19 September 2018	Frosta, Trondheimsfjord	63.5656	10.3019	WP2 180 μm	0–200			This study
26 April 2019	Raunefjorden	60.2730	5.1938	Dip net	0–100			This study
27 April 2019	Raunefjorden	60.2699	5.2208	Dip net	Surface			This study
Additional observations								
13 April 2011	Gullmarsfjorden, Sweden	58.2979	11.4917	Observation				GBIF/Artportalen
17 March 2013	Saltstraumen, Bodø	67.2276	14.6244	Observation				GBIF/Vebjørn Karlsen
17 March 2014	The White Sea, Kandalaksha Bay, Russia	66.5300	33.1000	eDNA				GBIF/White Sea Picoplankton metagenome
26 July 2016	Svalbard	79.0517	11.1075	eDNA				GBIF/MGnify
30 July 2016	Svalbard	80.6557	22.0855	eDNA				GBIF/MGnify
30 July 2016	Svalbard	80.6557	22.0855	eDNA				GBIF/MGnify
30 July 2016	Svalbard	80.6557	22.0855	eDNA				GBIF/MGnify
28 April 2018	Stora Leskär, Sweden	58.3751	11.2111	Observation				GBIF/Artportalen
NA	The White Sea, Russia	NA	NA	Diver observation				Alexader Semenov
*Mertensia ovum*								
21 August 2015	Svalbard	80.68533	15.5315	Juday 180 μ	0–470	MT614571	N	This study
21 August 2015	Svalbard	80.68533	15.5315	Juday 180 μ	0–470	MT614587	O	This study
09 July 2016	Svalbard	78.09276	13.55713	WP3 1000 μm	0–200	MT614568	P	This study
09 July 2016	Svalbard	78.09276	13.55713	WP3 1000 μm	0–200	MT614570	Q	This study
10 July 2016	Svalbard	77.42011	14.42702	WP3 1000 μm	0–120	MT614585	R	This study
10 July 2016	Svalbard	77.42011	14.42702	WP3 1000 μm	0–120	MT614580	S	This study
10 July 2016	Svalbard	77.42011	14.42702	WP3 1000 μm	0–120	MT614586	T	This study
11 July 2016	Svalbard	77.409	14.267	WP3 1000 μm	0–140	MT614584	U	This study
11 July 2016	Svalbard	77.409	14.267	WP3 1000 μm	0–140	MT614588	V	This study
11 July 2016	Svalbard	77.31926	14.38762	WP3 1000 μm	0–45	MT614581	W	This study
13 July 2016	Svalbard	76.555	15.143	WP3 1000 μm	0–190	MT614569	X	This study
13 July 2016	Svalbard	76.555	15.143	WP3 1000 μm	0–190	MT614567	Y	This study
13 July 2016	Svalbard	78.1008	13.4708	WP3 1000 μm	0–250	MT614572	Z	This study
17 July 2016	Svalbard	78.1008	13.4708	WP3 1000 μm	0–250	MT614578	AA	This study

^*^ = not available.

Sample processing varied between the sampling events. In general, specimens were gently sorted from the rest of the plankton sample immediately after collection and were counted. Selected specimens were photographed (macro photo or camera attached to a stereo microscope) and were examined under a stereo microscope alive prior to individual fixation in >70% ethanol for molecular analysis. Oral–aboral length was measured from live specimens or from photographs with a size-scale.

### Further observations

Observations were also obtained by accessing Global Biodiversity Information Facility (GBIF) data on *Euplokamis*, searching the web for the underwater images of *Euplokamis* spp. in Norway and soliciting help from underwater photographers (Table I). Photographic documentation was examined to identify *Euplokamis* specimens to species level.

### Molecular data

In total, 13 specimens morphologically identified as *Euplokamis* spp. were selected for molecular analysis. Additionally, 14 randomly selected *Mertensia ovum* (Fabricius, 1780) specimens collected from north of Svalbard in August 2015 and west coast of Svalbard in July 2016 were selected for molecular analysis in order to calculate the intra- and interspecific variations more accurately. DNA was extracted from tissue with a modified Chelex rapid-boiling procedure (Granhag *et al.*, 2012). 18SrDNA (approximately 1600–1800 bp) amplifications were performed on an MJ Research PTC 100 Thermal Cycler PCR with universal eukaryotic primers for 18S rDNA (Kober and Nichols, 2007) as explained in Granhag *et al.* (2012). PCR products were purified using Illustra GFX PCR DNA and gel band purification kit, following the cleaning procedure recommended by the manufacturer. Cycle sequencing of the PCR products was carried out by Macrogen Sequencing Service (Macrogen Inc, South Korea). The resulting nucleotide sequence electropherograms were checked by eye for poor base calls and sequence quality using Chromas Lite 2.1 (Technelysium Pty Ltd). The good-quality sequences were assembled using BioEdit software (Hall, 1999).

To place our sequences phylogenetically, all available complete 18S rDNA sequences of Ctenophora, and four Cnidaria sequences as an out-group, were retrieved from the NCBI nucleotide database (GenBank, accessed 13 September 2019). Additionally, four specimens collected by Granhag *et al.* (2012), of which three have been published earlier for ITS1 and partial 18S rDNA sequences (HE805699, HF912430 and HE805698), were reanalyzed for complete 18S rDNA sequences. Sequences from GenBank were combined with our sequences and aligned with the MAFFT online service (Katoh *et al.*, 2019), using the Q-INS-i strategy accounting for RNA secondary structure, gap-opening penalty of 1.53 and gap extension penalty of 0.123. The alignments were visually checked, non-alignable regions were removed (85 bp) and identical sequences were excluded prior to the analyses. The final 18S rDNA alignment contained 88 variable ctenophore sequences with 1663 bp, 1237 bp of which were constant, 426 variable and 303 parsimony-informative. Five sequences (two GenBank sequences and three from this study) were 24–611 bp shorter and question marks were added in the beginning or the end of these sequences. For the alignments see Supplementary materials 1 and 2 (see online supplementary data).

Bayesian phylogenetic analysis was performed with MrBayes 3.2.7a (Ronquist *et al.,* 2012). Two independent runs with four Markov chains and 1600 000 generations were carried out [average standard deviation (SD) of split frequencies 0.0069]. The sampling was conducted across the GTR model space with gamma-distributed rate variation across sites and a proportion of invariable sites, and the resulting estimates (e.g. tree topology) were used as posterior probability weighted averages of the models. Maximum likelihood bootstrap support values were calculated from 1000 replicates, using GARLI 2.0.1019 (Zwickl, 2006) with jModelTest 0.1.1 (Posada, 2008) AICc criterion selected model (TIM2 + I + G). The sequences reported in this paper have been deposited in the European Molecular Biology Laboratory (EMBL) nucleotide sequence database (MT614564–MT614590).

Intrageneric 18S rDNA variation of *Euplokamis* (HE647719, MF599307, sequences from this study) as well as intraspecific variation of *M. ovum* and *Pleurobrachia pileus* (O. F. Müller, 1776) (all publicly available sequences and additional *M. ovum* sequences from this study) were determined by the K2P method and the p-distances were determined by using MEGA X (Kimura, 1980; Collins *et al.*, 2012; Srivathsan and Meier, 2012; Čandek and Kuntner, 2015; Kumar *et al.*, 2018). Both transition and transversion substitutions were included; with gamma distributed (G) selection in rates and sites option with number of discrete gamma categories set as 5 and with 95% site coverage cut-off. Intrafamily divergence for Mertensiidae and Pleurobrachiidae was similarly determined for comparison.

## RESULTS

### Geographical and vertical distribution

**Fig. 2:**
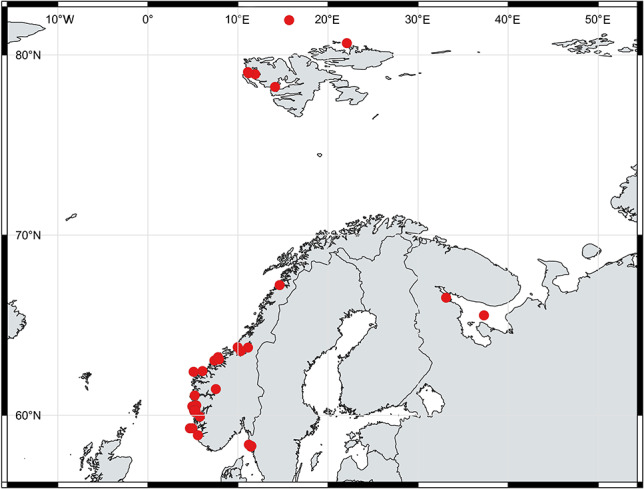
*Euplokamis* observations from the Norwegian coast including Svalbard region, and adjacent waters.

The data combined for this study show that ctenophores of the genus *Euplokamis* have been observed along large parts of the Norwegian coast, from southern Norway to Bodø and around the Svalbard archipelago, including north of Svalbard, to almost to 82°N (Fig. 2, Table I). In adjacent waters, *Euplokamis* spp. has been reported both from the White Sea in the north as well as the Swedish west coast in the south. The genus occurs inside fjords as well as offshore. Collection of specimens from known depths, with dip nets from the surface and during depth-stratified net sampling with Multinet and MOCNESS, suggests a wide depth distribution from the surface down to 100 m (Table I). One individual was also recorded from depth-stratified Multinet sample from 500 to 1000 m. However, the exact collection depth for many net-collected specimens is not known, as a single tow may cover a large portion of the water column. Diver observations generally come from the upper 30 m of the water column. The compiled observations from Norwegian waters start in 1999. Most of the observations are from between April and July, whereas some specimens have been found in March as well as in October–December.

Out of the 50 worldwide records of the family Euplokamididae in GBIF, only three are identified to species level, as either *E. dunlapae* or *E. stationis* [GBIF.org (accessed 22 January 2020)]*.* Of these 50 GBIF records, three are from Norwegian waters and a further three from adjacent areas (Table I), and all were identified as *Euplokamis* sp.

### Species identity

The net-collected specimens were identified as *E. dunlapae,* whereas specimens with only photographic ID where identified as *Euplokamis* sp. Our net-collected specimens are morphologically mostly similar to *E. dunlapae*, as described by Mills (1987), with respect to the body shape and length of the comb rows, and three of the *Euplokamis* 18S rDNA sequences from our study were identical to an *E. dunlapae* sequence from the vicinity of the type locality in Friday Harbor, USA (MF599307). However, the observed intrageneric variation of *Euplokamis* was higher than the intraspecific variation of *M. ovum* and close to the intrafamily divergence of Pleurobrachiidae (Table II). While this may suggest the potential hidden diversity within the analyzed sequences, no geographic structuring for the observed diversity was evident.

**Table II:** 18S rDNA intra- and intertaxon divergences (%, K2P and p-distances) of cydippid ctenophores common in the study area

Kimura	Average	SD	Min	Max
*Mertensia ovum* (*n* = 11)	0.07	0.09	0.00	0.33
*Euplokamis* sp. (*n* = 10)	0.21	0.09	0.00	0.43
*Pleurobrachia pileus* (*n* = 2)	0.11	NA	NA	NA
Mertensiidae (*n* = 17)	1.22	1.48	0.00	3.78
Pleurobrachiidae (*n* = 10)	0.28	0.21	0.00	0.76
*Mertensia ovum* versus *Euplokamis dunlapae*	0.35	0.09	0.22	0.65
*Mertensia ovum* versus *Pleurobrachia pileus*	5.15	0.09	5.10	5.35
*Euplokamis dunlapae* versus *Pleurobrachia pileus*	5.48	0.09	5.35	5.60
*P*-value	Average	SD	Min	Max
*Mertensia ovum* (*n* = 11)	0.08	0.09	0.00	0.19
*Euplokamis* sp. (*n* = 10)	0.21	0.09	0.00	0.25
*Pleurobrachia pileus* (*n* = 2)	0.11	NA	NA	NA
Mertensiidae (*n* = 17)	1.16	1.40	0.00	3.59
Pleurobrachiidae (*n* = 10)	0.27	0.21	0.00	0.68
*Mertensia ovum* versus *Euplokamis dunlapae*	0.35	0.09	0.22	0.65
*Mertensia ovum* versus *Pleurobrachia pileus*	4.79	0.09	4.75	4.96
*Euplokamis dunlapae* versus *Pleurobrachia pileus*	5.07	0.08	4.96	5.18

### Morphology

The most characteristic morphological feature of *Euplokamis* spp. is the coiled tentilla on the tentacles, giving the tentacle a beaded appearance when viewed from a distance (Fig. 1). Unfortunately, the tentacles were often damaged during net sampling and could not be used to identify to the genus level. This is, however, an excellent character to reliably identify the genus from the underwater photos or video footage of live specimens (cf. Neitzel *et al*., personal communication) and is helpful for evaluating the photographic evidence of occurrence.

All net-collected specimens during this study were elongate or ovoid in general appearance; in cross-section, cylindrical or slightly compressed in the stomodaeal plane (Fig. 1, Table I). Oral–aboral length of the measured specimens was <2–12 mm, but some of the specimens observed by the divers had a more elongate morphology, suggestive of a larger size. Large specimens were more elongated and had more prominent short keels projecting beyond the apical organ. Both adult and juvenile specimens had transparent, bluish mesoglea with conspicuous muscle fibers. Red pigmentation was present as rows of distinct patches on either side of the comb rows and on the tentacle bases, while the coiled tentilla appeared pinkish. The younger individuals in particular also had reddish pigmentation in the apical organ. The comb rows extended from two-third to three-fourth of the body length and had relatively large, tightly packed comb plates. The length of the cilia in the comb rows was relatively longer for small individuals, giving them a “furry” appearance (Fig. 1) that differs from the cydippid stage larvae of e.g. lobates, *Mnemiopsis leidyi* (Agassiz, 1865) and *Bolinopsis infundibulum* (Müller, 1776) as well as larvae and small individuals of *P. pileus* and *M. ovum* (Cydippida) also present in the study area. Tentacle bulbs, parallel to the stomodeum, became progressively more elongated with size and were located toward the oral end in the smaller specimens and more centrally in large specimens. The tentacle sheaths opened aborally and tentacles (when undamaged) carried the characteristic, widely spaced and tightly coiled side branches. Mouth was frequently observed protruding, particularly in the smaller specimens. This might, however, be due to collection damage—the mouth of *E. dunlapae* has been described as “quite prehensile” (Mills, 1987), but it also appears to be easily damaged or deformed during net sampling.

### Molecular identification

**Fig. 3:**
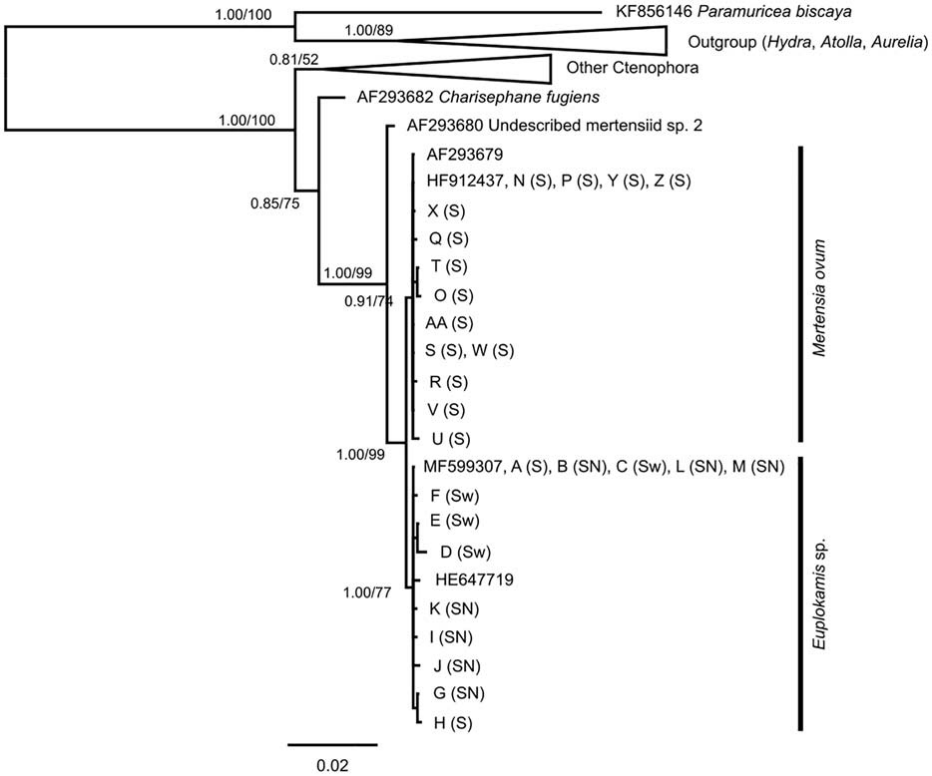
Maximum-likelihood tree for 18S of all ctenophore sequences in GenBank including the maximum likelihood bootstrap (TIM2 + I + G in Garli) and Bayesian posterior probability values (GTR + I + G in MrBayes). The letters indicate specimens sequenced in this study, see Table I for more information. Specimens with sequence ID HF912430, HE805698 and HE805699 are excluded from the analysis due being only partial 18S sequences. Letters inside the parenthesis indicate the sampling location: S, Svalbard; SN, southern Norway and Sw, Sweden. The tree was rooted with *Aurelia aurita* (Linnaeus, 1758), *Atolla vanhoeffeni* (Russell, 1957), *Hydro viridissima* (Pallas, 1766) and *Paramufricea biscaya* (Grasshoff, 1977) as the outgroup. Horizontal branch lengths reflect genetic distances among taxa.

All the 13 *Euplokamis* spp. specimens used for molecular species identification produced good-quality 18S rDNA sequences, including 9 variable sequences. In the phylogenetic analysis, all of these sequences clustered together with *Euplokamis* sp. from Sweden (HE647719) and with *E. dunlapae* from Friday Harbor, USA (MF599307) (Fig. 3). Five individuals sequenced in this study, including specimens collected from Svalbard to southern Norway as well as a reanalyzed specimen from Sweden, were 100% identical with *E. dunlapae* isolate collected from Friday harbor, USA (MF599307). However, none of the specimens were 100% identical with the *Euplokamis* sp. sequence from the Sweden (HE647719). Similarly, the 14 specimens morphologically identified as *M. ovum* produced 14 good-quality 18S rDNA sequences, including 10 variable sequences. All these sequences clustered together with *M. ovum* (HF912437 and AF293679) from Svalbard.

18S rDNA successfully differentiated between the genus *Euplokamis* and the closest neighbor in the tree, *M. ovum—*a common cydippid in the Norwegian high Arctic (see Discussion; Table II). The intraspecific K2P divergence was 0.21 ± 0.09% (average ± SD) for specimens clustering as *Euplokamis* sp. and 0.07 ± 0.09% for specimens clustering as *M. ovum,* while the average K2P distance between the species was 0.35 ± 0.09% (Table II). Observed divergences were even more conspicuous between *Euplokamis* sp. and the other common cydippid in Norwegian waters, *P. pileus* (Table II). The p-distances between the sequences were similar to the K2P distances (Table II).

## DISCUSSION

Based on the observations collected for this study, it is evident that *E. dunlapae* is widely distributed in Norwegian waters and Svalbard, from south to north and from fjords to the open ocean. In contrast to some of the more commonly reported ctenophores from the area—such as *B. infundibulum*, *M. leidyi*, *P. pileus* and *Beroe* spp., *Euplokamis* cf. *dunlapae* appears not to form dense blooms. Individual specimens are nevertheless frequently encountered in plankton samples as well as observed by the divers in the region. Video-transects filmed during a recent cruise to the Norwegian Sea also revealed *Euplokamis* spp. to be a common midwater gelatinous predator in the area (Neitzel *et al*., personal communication).

We have identified the net-collected specimens from Norwegian waters as *E. dunlapae*  Mills, 1987. However, morphological identification of ctenophores can be challenging, both due to the lack of identification literature and the damage to specimens resulting from net sampling and sample processing. Ctenophores are exceedingly difficult to preserve, meaning that type specimens are generally not available for examination. There is also considerable undescribed diversity within the phylum (Haddock, 2004). The genus *Euplokamis* can be distinguished from all other ctenophores by the presence of cross-striated muscle filaments in the side branches of the tentacles, but this is not a useful feature for field identification. The resulting characteristic coiled tentilla, however, makes it easy to tell *Euplokamis* spp. specimens apart from other cydippid ctenophores, including those commonly occurring in Norwegian waters: *M. ovum* and *P. pileus*. If tentacles are not present, as is often the case with net-sampled specimens, these species also differ in their general body shape: the *Euplokamis* specimens in this study had an ovate or elongate (larger length-to-width ratio), only slightly compressed body (Fig. 1), whereas *M. ovum* is strongly compressed in the sagittal plane, and *P. pileus* of the same size class is almost spherical (Majaneva, 2014). In contrast to both *E. dunlapae* and *M. ovum, P. pileus* lacks red pigmentation. *Pleurobrachia pileus* also lacks keels, while two short gelatinous keels in the aboral pole were distinguishable for larger *Euplokamis* sp. specimens in our study (cf. large specimens in Mills, 1987). It should be noted that while the elongate body shape can be used to rule out *M. ovum* or *P. pileus*, it is not enough to identify a specimen from Norwegian waters as *Euplokamis* cf. *dunlapae*: an undescribed cydippid species with similar size and general body shape is also known to occur in the area (Hosia and Båmstedt, 2007). However, this undescribed cydippid has highly extensible tentacles lacking the coiled tentilla typical of *Euplokamis*, a statocyst located at the bottom of a short funnel, and in undamaged specimens, prominent horns surrounding the mouth (Hosia and Båmstedt, 2007; A. Hosia, S. Majaneva and H. Ringvold, personal communication). While it is possible to separate *Euplokamis* from the other cydippid ctenophores known to occur in Norwegian waters, the morphological variation within the genus and its species remains poorly studied and documented, both locally and globally.

On the molecular side, the small subunit (18S) ribosomal RNA gene has proved to be a useful marker for phylogenetic reconstruction and molecular identification at various taxonomic levels for several eukaryotes (e.g. Zimmermann *et al.*, 2011) but is known to be highly conserved among ctenophores (Podar *et al.*, 2001). Nevertheless, it is the marker with the largest number of publicly available ctenophore sequences in terms of species coverage as well as number of specimens per species. Public databases currently include a very limited number of any *Euplokamis* sequences, with only one *E. dunlapae* specimen identified at the species level, thereby rendering intrageneric comparisons impossible. Specimens sequenced in this study from the North Sea, west Norwegian fjords and Svalbard as well as previously published specimens from the Swedish west coast (HE647719, Granhag *et al.*, 2012) were found to match with the published *E. dunlapae* 18S sequence from the type locality in from Friday harbor, USA (MF599307).

Even though 18S rDNA is highly conservative among ctenophores and not necessarily suited for species-level identification (Podar *et al.*, 2001; Alamaru *et al.*, 2017), it appears to successfully differentiate between genera, including *Euplokamis* and *Mertensia* in this study (Fig. 3, Table II). In Alamaru *et al.* (2017), the average p-distance between the species in the benthic ctenophore family Coeloplanidae was 0.03 ± 0.007% south-east, ranging between 0.0 and 0.21%, and the average p-distance between genera (i.e. *Coeloplana* vs. *Vallicula*) was 1.5 ± 0.03% south-east. Our study shows intraspecific distances for *M. ovum*, *P. pileus* and *E. dunlapae* to be on average 0.08 ± 0.09, 0.11 and 0.21 ± 0.09%, respectively (Table II). Regarding species delimitation, it is interesting to note the close sequence similarity between *M. ovum* in the Arctic and a yet undescribed mertensiid species (AF293680) which inhabits the tropics (Podar *et al.*, 2001). These two mertensiid species only differ by a few nucleotides at the level of the 18S rDNA genes, although anatomically they are quite distinct. The p-distance for these two species is 0.6%, much higher than for among Coeloplana species, demonstrating that 18S rDNA could be used for accurate species identification marker for some taxa, but not all, and that it is currently not possible to determine a consistent level of between-species divergence for the marker within Ctenophora. To identify the suitability for species-level identification for specific taxa, further analyses with several specimens from multiple species would be needed.

While COI sequences show promise for ctenophore species identification (Alamaru *et al.*, 2017), there are currently publicly available COI sequences for only seven pelagic ctenophore species, of which only five are formally described (*Beroe ovata*, *Beroe cucumis*, *Beroe gracilis*, *M. leidyi* and *P. pileus*) and two new species are implied in Johansson *et al.* (2018) (*Beroe norvegica* and *Beroe anatoliensis*). There are also few sequences per species and, thus, limited information on variability. At the same time, the current published protocols for ctenophore COI sequencing seem suitable for only a limited number of species, with *Euplokamis* spp. not being one of these (S. Majaneva, personal communication). The internal transcribed spacer (ITS) regions are further markers used for barcoding and show potential as useful markers for reconstructing high-level relationships within ctenophores (Simion *et al.*, 2015). However, when ITS1 region sequences from some of the *Euplokamis* specimens collected during this study were analyzed with all publicly available pelagic ctenophore ITS1 sequences, the marker appeared to be insufficient to distinguish between *M. ovum* and *Euplokamis* sp. (Johansen, 2019), thus limiting the accuracy of species identification.

While lacking substantial previous records from the north-east Atlantic, *E. dunlapea* is considered a common midwater ctenophore in other regions, even though rarely caught in net surveys. Along the US west coast, *E. dunlapae* is frequently observed from submersibles in densities up to 10 ind per m^3^, while not present in the concurrent plankton tow samples from the same area (Mackie and Mills, 1983; Mackie, 1985). The recent observations by Neitzel *et al*. (personal communication) suggest a similar pattern for the Norwegian Sea. *Euplokamis* DNA has also been observed by several metabarcoding projects in the North Atlantic and Arctic waters [e.g. uncultured eukaryotes targeted locus (loci) in the Gulf of Maine: 4 occurrences (MGnify, 2019a), Amundsen Gulf Overwintering Eukaryote Community: 24 occurrences (MGnify, 2019b) and Arctic microbiome along Svalbard cross-shelf transects: 4 occurrences (MGnify, 2019c)], while the genus appears not to be recorded by standard plankton surveys from these areas. These examples are consistent with the comparisons between traditional net sampling and observations from optical platforms, which have shown that ctenophore diversity and abundance—and consequently, their ecological importance—are consistently underestimated when using net sampling, particularly in combination with formalin fixation (Hosia *et al.*, 2017). Mills (1987) also concluded that *E. dunlapae*, like most ctenophores, do not preserve well in fixed samples and are thus challenging to observe in standard plankton surveys.

Most of the *Euplokamis* cf. *dunlapae* observations in this study were collected between April and July, whereas some specimens were recorded in March as well as October–December. Spring observations were from southern or mid-Norway, whereas the first observations from northern Norway and Svalbard region were from July onward. This could, however, reflect the distribution of the sampling effort, as we have had no systematic sampling throughout the year. In Friday Harbor, *E. dunlapae* adults were most abundant during the spring and peaked in May, with larval specimens collected in July and September, while a series of submersible dives in Saanich Inlet, British Columbia, suggests a year around presence of the ctenophore (Mackie, 1985; Mills, 1987). Similarly, in a year-long time series of eukaryote community sampling using metabarcoding, *Euplokamis* sp. was recorded between February and July as well as in November and December in Amundsen Gulf, Arctic Ocean (MGnify, 2018b). Systematic sampling throughout the year would be required to confirm the seasonality and depth distribution of *E. dunlapae* along the Norwegian coast.

The observations collected for this study suggest the presence of *Euplokamis* cf. *dunlapae* from the surface to below 500 m depth in Norwegian waters. However, it is important to note that the exact collection depth for many net-collected specimens is not known, as a single net tow may cover a large portion of the water column. *Euplokamis dunlapae* is generally considered a midwater ctenophore, reaching its highest abundances below 250 m in the north-east Pacific (Mills, 1987; Mackie *et al.*, 1988) and between 100 and 112 m in the Swedish coast (Granhag *et al.*, 2012). Yet, observations from the surface waters close to shore occur as well (personal communication in Granhag *et al.*, 2012; P. Licandro, personal communication, this study), perhaps related to the upwelling events or mixing of the water column (e.g. Mills, 1987). *Euplokamis stationis* occurring in the Mediterranean was likewise found to be relatively common between 200 and 600 m in the Alboran Sea (Mills, 1996), despite being only rarely reported by other studies.

Despite scant earlier reports, we suggest that *E. dunlapae* is a relatively common, likely indigenous ctenophore along the entire Norwegian coast, including Svalbard. The conspicuous lack of records is probably attributable to the methodological constraints detrimental for estimating ctenophore diversity and abundance, such as routine net sampling and formalin preservation of samples as well as lack of taxonomical expertise on gelatinous zooplankton and the absence of the genus from commonly available identification literature. The previous scientific observations sited in this study stem from a few projects and researchers focusing on gelatinous zooplankton, while the extensive ongoing and historic plankton monitoring programs in Norwegian waters have produced no records of the species. The increasing number of amateur and professional UW photographers during the past decades has also contributed to an increase in the observations on genus *Euplokamis* as well as other gelatinous zooplankton (e.g. Oliveira, 2007; Hosia and Falkenhaug, 2015). Minor modifications to sample processing routines, such as introducing standardized photographs of live net samples prior to fixation, could significantly improve the potential of standard plankton surveys for also monitoring the diversity and abundance of ctenophores and other gelatinous zooplankton. Molecular methods such as eDNA and metabarcoding could also serve to increase the available data on ctenophore diversity and distributions in Norwegian waters, but they still require work on identifying suitable genetic markers and for building reference databases before becoming a fully feasible option.

## CONCLUSIONS

The commonly used net-based methods for plankton monitoring, particularly in combination with fixation of samples, are poorly suited for sampling ctenophores and lead to an underestimation of their abundance and diversity (Hosia *et al.*, 2017). Using a variety of data sources, including diver observations, we show that ctenophores belonging to the genus *Euplokamis* are more common in Norwegian waters than previously assumed. While the documented specimens are morphologically identified as *E. dunlapae* and the 18S sequences of several specimens are likewise identical with the *E. dunlapae* isolate originating from close to the type locality at Friday harbor, USA, it should be noted that morphological and molecular variation within the genus and its species remain poorly studied and documented.

## Supplementary Material

Majaneva_et_al_Euplokamis_Postfilterest_ForEuploFinalAln_fbab012

Majaneva_et_al_Euplokamis_Prefilterest_Original_Euplo_18S_fbab012
